# Monthly Summary

**Published:** 1887-07

**Authors:** 


### Monthly Summary.

---

Proximal Decays.—My method of treating proximal decays is to space the teeth apart considerably with cotton, wedging them twenty-four to forty-eight hours, and then contour them boldly, bracing the teeth firmly against one another, and if possible increasing the size of the arch, and bridging the amalgam or filling used across from one to another, almost amounting to bridge-work, with amalgam, going thus to the other extreme of that heroic cutting recommended by Dr. Darby. Teeth that are alive I have no solicitation about. They are cast out in nature's own time and in her own way, as the leaves drop from the trees in autumn.

In my early practice I used to pride myself on being very handy with forceps to aid nature in getting rid of deciduous teeth or roots, and conscientiously thought I was doing my patients good, by cleaning up their mouths in such manner as to have them look clean at least, when in reality I was doing them irreparable injury. I can now look over a set of teeth where the remains of the old ones are sandwiched between the new, and know that I am doing my patient a great benefit by leaving all of these unsightly looking pieces in the jaw, but I grind them smooth with corundum wheels, removing the sharp points and shorten them down sufficiently to prevent contact with the teeth of the occluding jaw.—*Dr. Morrison, in The Archives of Dentistry.*

---

A Method of Testing the Vitality of a Tooth's Pulp.—To determine whether a tooth's pulp is living or dead is the first question to be decided in the diagnosis of every case of odontalgia, the dentist is called upon to treat. Many obscure cases present in which this is a difficult point to ascertain, as occasional errors in the practice of the most careful will witness. Therefore any improvement in method which will facilitate the detection of the pulp's condition and render the result more certain, will be appreciated by all. A simpler and more efficient method of applying heat than that usually employed for this purpose, was suggested at the April meeting of the Odontological Society. It is as follows: The preliminary isolation and drying of the suspected tooth and the immediately adjoining ones is the same as by the old method, although the rnbber dam is not so essential. The tooth is then tested by applying to it a piece of gutta percha which has been heated over a flame. It takes hold and transmits its heat at once and there is an almost immediate response if the pulp is living. If there is no response, it may, without being again heated, be applied to one of the adjoining teeth, with a known living pulp, and the comparison noted. The old method of testing with a heated steel instrument is terrifying to a nervous patient and the response if the pulp be alive is often tardy in coming or may be entirely absent. Apprehensive patients under the influence of fears or imaginings will frequently, when a heated instrument is applied, mislead the operator by declaring that a tooth is sensitive when you know it to be pulpless.—*Dental Review.*

---

DR. RICHARDSON recently lectured before the Society of Arts (London) on the painless extinction of life in the lower animals. At the Dogs' Home it appears that over 6,000 dogs have been painlessly killed duiing the past seven months. The principal agent used for the narcotic action is carbonic oxide, passing, at 80° Fahr., over a mixture of chloroform and carbon bisulphide into a lethal chamber, in which chamber as many as 100 dogs can at once receive euthanasia. Dr. Richardson described a small apparatus which, after long trials, he has completed, in which from one to six animals can be painlessly killed, and which is so portable that it can be wheeled from a central station to any house or street ready for immediate use. By an extension of the same design the author next intends to apply it to animals of the larger kind that are used for human food, its application to the slaughtering of sheep being already quite feasible and inexpensive. Why could not a similar apparatus be substituted for the hangman's rope? Already a commission has been appointed to investigate and report to the Legislature of the State of New York the most humane and practical method known to modern science, of carrying into effect the sentence of death in capital cases, by which it would seem that the subject is commanding some attention.

---

COMPOSITION FOR DUPLICATING MODELS.—As I have much improved the duplicating composition which was brought under the notice of the Society two years ago, I have much pleasure in forwarding a sample for the members' inspection, also a model and place, showing the perfect accuracy of the duplicate. In working the composition, I found that it absorbed water and tended to impoverish the material and destroy the perfectness of the duplicate obtained. After trying several varnishes, and finding each defective, I think I have obtained the desideratum in a solution of india-rubber and benzoine. The solution is floated for a moment over the composition, and the surplus instantly poured off, and there is left behind an almost imperceptible coating, which is absolutely waterproof. As it now stands, I think it is perfect, and would eel pleased by members trying the composition and varnish, and at next meeting giving an expression of opinion.—*Whitehouse in Dental Record.*

---

DENTISTRY RECOGNIZED BY THE AMERICAN MEDICAL ASSOCIATION AS A SPECIALTY OF MEDICINE.—While we have held all the time that dentistry is a specialty of medicine, yet, this formal declaration of the American Medical Association lifts a barrier that has existed since a separate degree was created, and separate schools established. The medical profession has, of late years, shown a liberal spirit toward dentistry, as represented by its leading men. In their Congresses they made room for us, only, however, under the title of M. D.; and now, while the D. D. S. is recognized, it is only under a higher order of its acquirement that it is to be so. It now remains to be seen if our colleges will pull up to it or treat the recognition, as some individuals will do, unsought and unwanted.

While the opportunity did not wait for the full fruition of our plan, yet we find that this is a glorious epoch in our history. We must now consider the question of "A Specialty" as settled, and it is for us to move up to yet higher attainments. By carrying out the requirements of the resolution we shall occupy the same relation to medicine in this country as surgery does to medicine in England.

Dr. N. S. Davis, President of the International Medical Congress, to whom we are indebted for this resolution, states very clearly his reasons for doing so, and what he hopes to see it accomplish. The manner in which the resolution was received is certainly strong evidence of the estimate placed on dental surgery by the medical profession of America.

Dr. Allport, president of the American Dental Association, is also entitled to much favor for his work in the matter.—*Southern Dental Journal.*

---

GOLD SOLDER.—Dr. McKellops recommends that 89 parts of the plate used (be its carat what it may) be alloyed with 7 parts of silver and 4 parts of copper. This will always prove reliable, flow easily, and most nearly preserve the color of the plate. Should it be necessary to solder *over* this solder, reduce it further in the same proportions, *i. e.*, using the solder itself as the gold to be reduced.—*Dental Register.*

---

OBTUNDING SENSIBILITY.—If you have never tried it, ask your next patient for whom you are excavating a sensitive tooth to hold his breath after inflating his lungs. This is often done, if you have observed by the patient involuntarily. We were struck with this means of enduring pain, by animals, when a boy.—*Ex.*

